# Reference production in Mandarin–English bilingual preschoolers: Linguistic, input, and cognitive factors

**DOI:** 10.3389/fpsyg.2022.897031

**Published:** 2022-09-29

**Authors:** Jiangling Zhou, Ziyin Mai, Qiuyun Cai, Yuqing Liang, Virginia Yip

**Affiliations:** ^1^Childhood Bilingualism Research Centre, Department of Linguistics and Modern Languages, The Chinese University of Hong Kong, Hong Kong, Hong Kong SAR, China; ^2^Department of Linguistics and Translation, City University of Hong Kong, Hong Kong, Hong Kong SAR, China

**Keywords:** bilingual reference production, input, frequency, amount of language exposure, working memory, cross-linguistic influence, Mandarin, English

## Abstract

Reference in extended discourse is vulnerable to delayed acquisition in early childhood. Although recent research has increasingly focused on effects of linguistic, input, and cognitive factors on reference production, these studies are limited in number and the results are mixed. The present study provides insight into bilingual reference production by investigating how production of referring expressions in the two languages of preschool bilingual children may be influenced by structural similarities and differences between the languages, frequency of referring expressions in maternal input, amount of exposure to each of the languages, and working memory capacity. Using two stories in the Multilingual Assessment Instrument for Narratives (MAIN), we examined character introduction and re-introduction in oral narratives of 4–6-year-old Singaporean bilingual children acquiring Mandarin Chinese and English (*n* = 21), and in child-directed speech of the mothers (*n* = 17). The children’s language exposure, executive function, and general bilingual proficiency were also recorded or directly tested through structured interviews with the parents or standardized assessments with the children. Data collection was conducted remotely in real time over a video-conferencing platform, supplemented by on-site audio recording to ensure sound quality. Results showed prolonged development in the production of felicitous REs for first mentions and over-reliance on overt marking of definiteness in our bilingual children. Mixed modeling revealed that frequency of felicitous REs in the input predicted children’s production of felicitous REs across languages and discourse functions, with a modulating effect of working memory. Overall, our findings are consistent with previous ones in that reference production is vulnerable in early Mandarin-English bilinguals in a multilingual society. This study also presents novel evidence that structural frequency in the input interacts with working memory in shaping patterns of reference production in bilingual children.

## Introduction

Reference is one of the core aspects of human communication. A variety of linguistic structures such as lexical noun phrases (NPs, e.g., the goat), demonstratives (e.g., this), and personal pronouns (e.g., she) can serve as referring expressions (REs). To produce felicitous REs, speakers must develop sensitivity to language-specific constraints at syntactic, semantic, and discourse-pragmatic levels as well as the cognitive ability of perspective taking.

To introduce a new referent into discourse—for instance, a fox known to the speaker but not shared by the listener, adult speakers prefer indefinite expressions (e.g., *There is a fox hiding behind the tree*), rather than definite ones (e.g., *The fox is hiding behind the tree*). A lengthy period of development has been documented in children before adult-like use of REs in extended discourse (e.g., narrative production), with significant developmental changes occurring after 7–10 years (Hickmann et al., 2015). Monolingual children under 5–6 years have been shown to overuse definite nominals. They produced a substantial number of NPs with a definite determiner in article languages like English (e.g., Hickmann et al., 1996) or used inappropriate bare nouns interpretable as definite in article-less languages like Mandarin (e.g., Min, 1994; Wu et al., 2015) in contexts where the intended referents were new and unknown to the listener. Apart from choice of elements inside the NP, adult speakers manipulate word order to mark the new/old distinction, preferring the “old-before-new” word order. Young children, however, exhibit a preference for ordering new information before old information (e.g., German: Narasimhan and Dimroth, 2008) or display no ordering preference (e.g., English: Chen and Narasimhan, 2018; Mandarin: Chen et al., 2020). Though not always the case, bilingual children have been reported to show uses of REs that differentiate them from monolingual children, producing non-target-like forms unattested in monolingual children (Zhou et al., 2021; Zhou and Yip, 2021), and using linguistic forms to overtly mark definiteness to an excessive extent (Aalberse et al., 2017).

Different attempts have been made to account for children’s late mastery of reference in extended discourse situations and differences among individuals, examining the role of cross-linguistic influence, language input, and cognitive capacities. Although several recent studies on reference production have investigated the effects of input and/or cognitive factors (e.g., Jia and Paradis, 2015; Lindgren et al., 2020; Serratrice and De Cat, 2020), these studies are limited in number and the results are mixed. Research adopting a multifactorial perspective to reference production is in its infancy. We attempt to bring different lines of research on reference development closer together by investigating reference production in bilingual children, considering influence of linguistic, input, and cognitive factors. Specifically, we studied bilingual preschoolers speaking Mandarin Chinese (hereafter Mandarin) and English as well as their parents in Singapore, a multilingual society where English and Mandarin are widely spoken. 74.3% of the resident population in Singapore are Chinese, most of whom use English (47.6%) or Mandarin (40.2%) as the most frequently spoken language at home, and 75.5% of the Chinese who speak English most frequently at home also use Mandarin as the second most frequently spoken language at home (Singapore Department of Statistics, 2020). Unlike English, a non-pro-drop language with dedicated morphology to express definiteness, Mandarin encodes definiteness *via* word order, discourse context, and optional use of functional items (e.g., demonstratives). The central question in this article is how linguistic, input, and cognitive factors interact and shape reference production in Mandarin-English bilingual preschoolers in a multilingual society.

Another innovative feature of this study is that we elicited child and adult discourse remotely over an audio-video platform in real time (online) in lieu of the traditional face-to-face methods, which were rendered less feasible, if at all possible, due to social distancing during the COVID-19 pandemic. Remote online assessment has been shown to yield results comparable to face-to-face methods in tests of intellectual abilities, vocabulary and comprehension with preschool and school-age children (e.g., Hodge et al., 2019; Kronenberger et al., 2021; Werfel et al., 2021). However, little has been reported on the feasibility and validity of eliciting narratives from young children through virtual meetings in real time. This study will provide valuable data for future comparison of referential strategies used by children between face-to-face and videoconference-based modalities.

In the following, we review studies on the linguistic, input, and cognitive factors involved in reference production respectively, and present the research goals and methods of the current study, followed by results and discussions on the relation between reference production and the three sets of factors in each of the target languages.

## Reference production: Linguistic, input, and cognitive factors

### Referential choice in Mandarin and English: Form, function, and acquisition

A speaker’s referential choice usually reflects their assumptions about the extent to which a referent is linguistically retrievable or cognitively accessible to the addressee (Ariel, 1990; Gundel et al., 1993). Referents that are deemed more accessible (e.g., receiving shared visual focus of attention, made prominent by preceding discourse environment, and bearing the thematic role of agent) are likely pronominalized, while referents with low accessibility tend to be denoted by nominals (Allen et al., 2008).

In a narrative context, referent accessibility is often discussed in association with discourse function; that is, whether the RE mentions a referent for the very first time (referent introduction/INTRO), maintains reference to an already mentioned referent (reference maintenance), or re-mentions a referent after focusing on a different referent in intervening utterances (re-introduction/Re-INTRO) (Hickmann et al., 2015). This study will focus on INTRO and Re-INTRO, both of which involve reference to an entity that is outside the addressee’s current focus of attention and have been found to pose greater challenges for children than reference maintenance (Wong and Johnston, 2004; Chen and Lei, 2012; Colozzo and Whitely, 2014).

Regarding referential forms, pronominals (i.e., demonstratives and personal pronouns), and null forms^1^ neither signal new information nor fulfill the dual purposes of signaling given information while acknowledging a topic shift (Colozzo and Whitely, 2014). They are more suitable for maintenance of reference, and less preferable than nominals for either INTRO or Re-INTRO. Definite nominals presuppose the listener’s knowledge whereas indefinite ones do not. Given this, it is natural that indefinite nominals are preferred in INTRO contexts to introduce new referents and definite nominals in Re-INTRO contexts to shift the topic and bring forward previously mentioned referents.

In both Mandarin and English, there are identifiable nominals which have interpretable reference (definite or specific) independent of syntactic position, such as demonstrative NPs (e.g., *zhe zhi yang* “this goat”), kinship terms (e.g., *mama “*mom”), and complex NPs containing a possessor [e.g., *ta (de) mama “*her mother”], a relative clause (e.g., *zai chi cao de yang* “the goat that is eating grass”), or adjectival modification (e.g., *niaochao li de xiaoniao* “the birds in the nest”). An interesting fact about Mandarin demonstrative NPs is that the distal demonstrative *na* “that” is arguably going through a grammaticalization process, in which it has developed additional functions that are typically served by definite articles in languages like English (Chen, 2004). For instance, unlike the demonstrative *na* in (1a), which expresses a distal meaning, *na* in (1b) is deictically neutral, serving as a determiner of a complex NP. This is also reflected by different translations in English in (1a) and (1b), where the demonstrative *na* is felicitously translated into *that* and *the, respectively.* Mandarin demonstrative NPs sometimes appear without the noun in the form of [demonstrative-classifier], functioning like a demonstrative pronoun as in (1c).

1a. Demonstrative NP used deictically.

Zhe/Na zhi yang hen ke’ai.

this/that cl goat very cute

“This/That goat is very cute.”

(Context: the speaker refers to a goat nearby/from a distance.)

1b. Demonstrative NP in deictically neutral contexts.

Yang mama faxian le na zhi duo zai shu houmian de huli.

goat mother discover le that cl hide at tree back de fox

“Mommy goat saw the fox that was hiding behind the tree.”

1c. [demonstrative-classifier] functioning like a demonstrative pronoun.

Zhe/Na ge shi shenme?

this/that cl is what

“What is this/that?”

While there are structures in which Mandarin and English overlap in both form and function, there exist language-specific structures for expressing definiteness. In English, definiteness is marked by definite/indefinite/numeral determiners distinguishing given from new referents [(Def./Indef./Num. determiner-NP); e.g., *the fox*, *a fox*, *and two foxes*]. Mandarin is an article-less language which does not have such a mechanism for denoting definiteness. Instead, definiteness marking is achieved through a number of nominal structures and their positioning in relation to their subcategorizing verbs. Regardless of RE type, new information typically appears postverbally in Mandarin. Below we will present two such structures [(bare noun) and (numeral-classifier-noun)] and show how their referential meaning changes when they appear pre- and postverbally.

Unlike in English, bare nouns are allowed in Mandarin and interpretable as definite or indefinite, depending on whether they are preverbal or postverbal (Cheng and Sybesma, 1999).^2^ Preverbal bare nouns tend to be definite, as shown by *huli* “the fox” in (2), whereas postverbal bare nouns such as *shanyang* “goat” in (2) tend to receive an indefinite reading, unless when they refer to already mentioned or known referents (e.g., in Re-INTRO contexts).

2. Bare noun used for INTRO contexts (definite preverbally, indefinite postverbally)

Huli xiang chi shanyang.

fox want eat goat

“The fox wanted to eat a goat.”

A second structure encoding (in)definiteness in Mandarin but not in English is the [Num-Cl-N] structure consisting of a numeral (Num), a classifier (Cl), and a noun (N), as shown by *yi zhi huli* “a fox” in (3). Like bare nouns, postverbal [Num-Cl-N] structures are interpreted as indefinite, unless in contexts where the intended referent is already mentioned and identified. Preverbal [Num-Cl-N] structures usually receive a definite interpretation, as shown by *liang zhi xiaoniao* “the two little birds” in (4a). The [Num-Cl-N] is indefinite when the numeral is *yi* “one” (i.e., [*yi*-Cl-N]) and the [*yi-*Cl-N] cannot be placed preverbally or used for Re-INTRO as in (4b).

3. Postverbal indefinite [Num-Cl-N] (numeral optional when it is *yi* “one”)

Yang mama kanjian (yi) zhi huli.

goat mother see one cl fox

“Mommy goat saw a fox.”

4. Preverbal definite [Num-Cl-N] (impossible when the numeral is *yi*)

a. Liang zhi xiaoniao kanjian xiaomao lai le, hen haipa. two cl little bird see little cat come le very scared “Seeing the little cat coming, the two little birds were very scared.”

b. *Yi zhi xiaoniao kanjian xiaomao lai le, hen haipa. one cl little bird see little cat come le very scared Intended: “Seeing the little cat coming, the little bird was very scared.”

In addition to appearing in canonical Subject-Verb-Object (SVO) sentences as in (2–3), both bare nouns and [Num-Cl-N] structures characteristically occupy postverbal positions in existential *you*-sentences and Subject-Verb (SV) inversion sentences to introduce new referents (Li and Thompson, 1981). This is consistent with their indefinite interpretation in the postverbal position, illustrated in (5) and (6), where *(yi zhi) huli* “(a) fox” appears after the existential verb *you* “have” and the motion verb *lai* “come” respectively. A summary of the nominal expressions used for INTRO and Re-INTRO contexts in Mandarin and English is given in Table 1.

**Table 1:** Nominal expressions and their discourse functions in Mandarin and English.

Nominal expression	Discourse function	Linguistic form	Position	Mandarin	English
Indefinite	[INTRO]	Bare noun^a^	Postverbal	+	N/A
		[Num-Cl-N]	Postverbal	+	N/A
		[Indef. determiner-NP]	Pre/postverbal	N/A	+
		[Num. determiner-NP]	Pre/postverbal	N/A	+
Definite/Identifiable	[Re-INTRO]	Bare noun	Pre-verbal	+	N/A
		[Num-Cl-N]	Pre-verbal	+	N/A
		[Def. determiner-NP]	Pre/postverbal	N/A	+
		Demonstrative NP	Pre/postverbal	+	+
		Other nominals with interpretable reference^b^	Pre/postverbal	+	+

“+,” allowed; N/A, non-applicable; Shaded cells are structures in which Mandarin and English overlap in terms of form and function.

a Bare noun and [Num-Cl-N] for first mentions of referents tend to be interpreted as indefinite postverbally and as definite preverbally. They receive a definite reading when referring to already mentioned referents. Referential [(yi-)Cl-N] is indefinite.

b Other nominals with interpretable reference regardless of syntactic position include kinship terms and complex NPs containing a possessor, a relative clause or adjectival modification.

5. Bare noun and [Num-Cl-N] in existential you-sentence.

You (yi zhi) huli duo zai shu houmian.

have one cl fox hide at tree back

“There is a fox hiding behind the tree.”

6. Bare noun and [Num-Cl-N] in SV inversion.

Lai le (yi zhi) huli.

come le one cl fox

“A fox came.”

The aforementioned differences in reference coding have been shown in adult Chinese/English speakers’ narrative production. In Hickmann et al. (1996), the Chinese speakers mostly used postverbal [Num-Cl-N] [e.g., (3, 5–6)] for INTRO, while the English speakers marked most of the INTROs with an indefinite determiner (likely postverbal, but less frequently compared to Chinese speakers). Hickmann and Hendriks (1999) found that most nominals denoting previously mentioned referents in Re-INTRO contexts were bare nouns [e.g., (2)] and demonstrative NPs [e.g., (1a)] in Chinese speakers and [Def. determiner-NP] in English speakers. These findings confirm that definite/indefinite determiners are the primary mechanism for marking the given/new distinction in nominals in English, while a number of morphosyntactic structures collaborate with word order to mark that distinction in Mandarin.

For children, previous studies showed that monolinguals overproduce definite nominals in English/Mandarin (Hickmann et al., 1996; Wu et al., 2015) and differ from adults by showing no preference for the “old-before-new” word order (Chen and Narasimhan, 2018; Chen et al., 2020). For Mandarin-English bilingual children, a question is how they cope with dual input in developing target-like reference use. Cross-linguistic influence, specific language input, and cognitive capacities have featured frequently in recent literature. We will review cross-linguistic influence in the rest of this section and the input and cognitive factors in the next two sections.

Cross-linguistic influence (CLI) is likely to take place in domains involving syntax-discourse interface when there is structural overlap between two languages being acquired by the bilingual child (Hulk and Müller, 2000). In this light, reference production is predicted to be vulnerable to CLI. Indeed, evidence for CLI has been reported in previous studies on bilingual children learning Mandarin and an article language. For example, Mai et al. (2021) found that heritage Mandarin children (aged 4–14) in the United Kingdom produced significantly more demonstrative NPs in a syntactic position requiring definite or specific NPs. The authors attributed this difference to possible CLI from English, which obligatorily marks definiteness through overt markers. In Aalberse et al. (2017), heritage Mandarin speakers (aged 15–27) in the Netherland also showed a significant increase in the use of demonstrative NPs in oral narratives, compared to homeland speakers. It was suggested that demonstrative pronouns in Mandarin might have been reinterpreted as definite articles by the heritage speakers due to influence of Dutch, which has dedicated morphology to encode definiteness. Both studies point toward CLI from the language with overt definiteness marking (English, Dutch) to Mandarin. Looking beyond Mandarin, the use of demonstratives as an equivalent of definite articles has been found in other article-less languages, such as Russian, Malay, and Polish in contact with article languages (Polinsky, 2006; Moro, 2016; Otwinowska et al., 2020). These findings are invariably consistent with possible influence of an article-language on an article-less language with respect to definiteness marking.

The above studies either investigated older school-age children or included children with a wide age span, and the target language was a minority language mainly spoken at home. It remains open whether Mandarin-English bilingual preschoolers in a multilingual society where both target languages are spoken would exhibit over-reliance on overt marking of definiteness in Mandarin and behaved similarly to monolinguals regarding pre/postverbal positioning for first mentions (i.e., INTROs), which brings us to input-related factors in bilingual referential choice.

### Language exposure and caregiver input in bilingual acquisition

Compared to monolingual children, the input available to bilingual children is proportionally less in each language and typically unevenly distributed across the relevant languages. In cases where the linguistic input is presumably provided by caregivers who are non-native speakers or speak a contact variety of the language, it may also differ from the input monolingual children typically receive in that language in terms of quality (Paradis and Navarro, 2003; Fernald, 2006). Even within bilingual children, the input varies both quantitatively and qualitatively (e.g., presence of school-age older siblings in the home, one or both parents are native speakers of the target language), leading to individual differences in the rate of language growth (Hoff et al., 2014).

Accumulating evidence shows an enormous impact of language input on acquisition outcomes across various linguistic domains (Grüter and Paradis, 2014). The role of input on bilingual reference production is however less clear. For bilingual children who spoke different L1s at home and were schooled exclusively in English in the UK (5-7-year-old), those with greater exposure to English were better at providing informative REs than those with less exposure to English (Serratrice and De Cat, 2020). For heritage speakers of Mandarin (6;9–10;10), those who arrived in Canada at an older age and had a richer and more diverse Mandarin environment at home demonstrated superior performance with INTROs in Mandarin (Jia and Paradis, 2015). Nevertheless, these results contrast with Lindgren et al. (2020), who did not find a significant effect of amount of language exposure on Swedish-German bilingual children (4;0–6;11) in their use of indefinite NPs to introduce referents in either language. It was hypothesized that the null effect of language exposure could be due to typological similarities between Swedish and German in the use of REs for INTRO and the children’s relatively high proficiency in both languages.

Note that these studies invariably measured input based on retrospective parental report on the amount and source of the input. Parental report is a valid method to document and calculate coarse-grained input variables (Paradis, 2017). However, it inevitably oversimplifies the picture, as what is actually heard by the children is not captured. A fine-grained transcript-based analysis of real-life child-directed speech would enable us to obtain a more precise understanding of the ways in which input influences children’s reference production. Few existing research on reference production has adopted a fine-grained approach to input. An exception is Paradis and Navarro (2003), who analyzed spontaneous language data from one Spanish-English bilingual child (1;9–2;6), two Spanish monolingual children (1;8–2;7 and 1;8–1;11) and their parental interlocutors. Among many fine-grained variables of the input, they measured structural frequency, which has been reported to positively correlate with acquisition of grammatical structures such as *wh*-questions, relative clauses, and passives (see Ambridge et al., 2015, for a review). They found that not only the bilingual child produced more overt subjects than the monolingual children in Spanish, but the parents of the bilingual child also used overt subjects at a higher rate than the parents of the monolingual children. The findings suggested that differential patterns in the bilingual children’s referential choice may be influenced by how often relevant structures are provided in the parental input. The potential effect of structural frequency warrants further investigations with a larger sample including children with different language profiles and their caregivers, which is a motivation of our study.

### Working memory and reference production

When telling a story, in addition to accessing appropriate lexical and syntactic forms, a speaker must attend to the target referent, monitor for differences in the addressee’s perspective, and integrate visual and verbal information into a coherent situation model; furthermore, they must maintain and update the situation model by retaining information associated with a discourse referent and retrieving and updating this information in subsequent mentions of the referent (De Cat, 2015). This is a complex set of operations requiring attentional resources and support of executive functions—higher-order cognitive skills for planning and executing complex tasks (Pennington and Ozonoff, 1996; Miyake et al., 2000).

In particular, working memory possibly underpins the use of REs by allowing for an interlocutor to store and update the addressee’s perspective and check for convergence by comparing it with one’s own perspective (Serratrice and De Cat, 2020). The hypothesis is that when the communication task generates excessive cognitive demand for the parser’s working memory, they revert to a more “egocentric” mode (Nilsen and Bacso, 2017) and produce inadequate REs. Nevertheless, the findings have been mixed as to the role of working memory in reference use. In monolingual populations, children, adolescents, and adults with weaker working memory capacity have been shown to encounter greater difficulty in perspective-taking (e.g., Lin et al., 2010; Wardlow and Heyman, 2016; Nilsen and Bacso, 2017). Additionally, computational modeling studies have found that a simulated low working memory model would produce significantly more underspecified REs than a high working memory model (van Rij, 2012; Hendriks, 2016). Further evidence of a positive correlation between working memory and reference production comes from Torregrossa (2017), who found that German monolingual children (8-10-year-old) with lower working memory capacity were less adequate in the production of demonstrative pronouns in oral narratives. However, in Nilsen and Graham (2009), working memory was not predictive of English-speaking children’s (4-5-year-old) use of disambiguating modifiers when there was shared access to a referential alternative.

Mixed findings have also been reported in studies on bilingual reference production. Serratrice and De Cat (2020) reported that working memory positively correlated with 5-7-year-old bilingual children’s ability to use informative REs for anaphoric reference in English in the presence of a discourse competitor. Torregrossa et al. (2021), however, did not observe any correlation between updating skill (which hinges on working memory) and the production of underspecified pronouns (null subjects and clitics) in Greek in an elicited narration task with Greek-Albanian, Greek-English, and Greek-German bilingual children (7-13-year-old).

Possibly the mixed findings were due in part to differences in the experimental design, operationalization of working memory, and/or age of the participants. Nilsen and Graham (2009) and Serratrice and De Cat (2020) studied preschoolers performing referential communication tasks, and measured cognitive skills by memory of objects and/or backward digit span (BDS). Torregrossa (2017) and Torregrossa et al. (2021) elicited oral narratives from school-age children, with cognitive skills measured by BDS and a 2-back task, respectively.

The interaction between the linguistic and cognitive abilities of the speaker is already particularly intricate (Hendriks, 2016). Such interaction between language and cognition in bilingual children is further complicated by factors such as input and language dominance. In Torregrossa et al. (2021), for example, children who were dominant in Greek produced more overspecified full nouns as a function of lower updating skills, but such effect was absent in children who were dominant in other languages. It was argued that the effects of updating skills were overshadowed by the effects of language exposure in these children, since dominant experience in other languages led to the same pattern of outcomes as lower updating skills in terms of the use of full nouns in Greek—that is, children who were more dominant in other languages showed a stronger tendency of using overspecified full nouns in Greek, regardless of updating skills.

**Figure 1:**
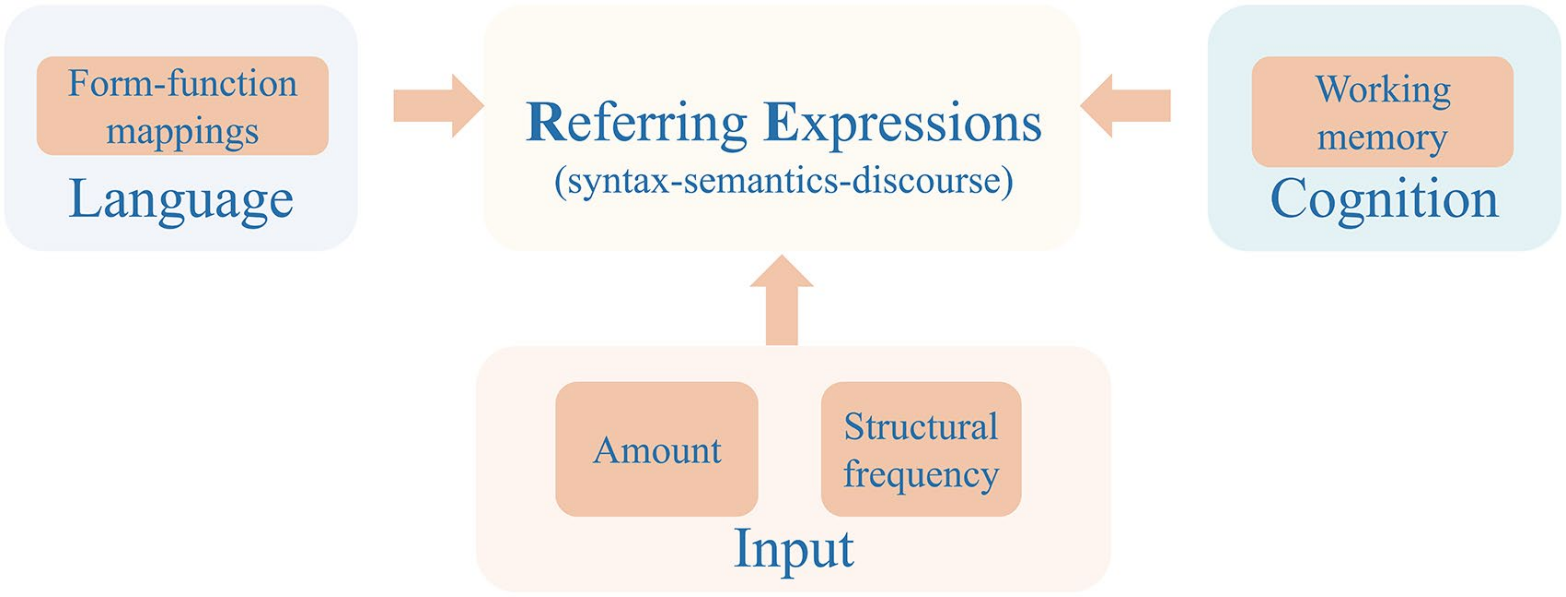
Research framework of the current study: Reference use and its relationship with linguistic, input, and cognitive factors.

Studies adopting a multifactorial approach, therefore, provide a window into the interplay between linguistic, input, and cognitive factors in bilingual reference production. The findings will shed light on the sources of bilingual-monolingual differences as well as individual differences in reference development. To this aim, we elicited narration from Mandarin-English bilingual preschoolers and collected child-directed speech data by recording storytelling by their mothers.^3^ We examined children’s production of REs at lexical, syntactic and discourse levels, and investigated its relations with maternal input (in terms of structural frequency), amount of language exposure, and working memory in each of the target languages. Figure 1 illustrates our research framework.

## The study

### Research questions and predictions

This study investigates 4–6-year-old Singaporean Mandarin-English bilingual children’s referential choice for INTRO and Re-INTRO in oral narratives, and examines the contribution of linguistic, input, and cognitive factors to bilingual reference production. Our specific research questions are:

#### Linguistic factors

What types of REs do Mandarin-English bilingual children use in INTRO and Re-INTRO contexts, respectively, in each target language? Do they show any position (preverbal, postverbal) preference for INTRO?

##### Predictions

Preferable REs for INTRO and Re-INTRO are indefinite nominals and definite/identifiable nominals, respectively. Considering the persistent overuse of inadequate REs in monolingual preschoolers shown in previous studies (Hickmann et al., 1996; Wu et al., 2015), we expect similarly non-target-like use of REs in our bilingual children—namely, over-production of [Def. determiner-NP] in English and NPs interpretable as definite in Mandarin in INTRO contexts.

Previous research showed over-reliance on overt markers to express definiteness in Mandarin heritage speakers due to cross-linguistic influence of English (e.g., Aalberse et al., 2017; Mai et al., 2021). If influence of English also occurs in our bilingual children, they will produce a high frequency of demonstrative NPs as older heritage Mandarin speakers in previous studies did.

English/Mandarin monolingual children were less likely to use the “old-before-new” word order than adults (Chen and Narasimhan, 2018; Chen et al., 2020). Given this, we expect no preference for postverbal INTROs over preverbal INTROs in either language of the bilingual children.

#### Input factors

How does Mandarin-English bilingual children’s reference production compare to the maternal input? Do they correlate in terms of structural frequency of REs? To what extent is Mandarin-English bilingual children’s referential choice influenced by the amount of exposure they receive in each target language?

##### Predictions

Mother–child differences are expected since the children are predicted to overproduce definite nominals for INTRO, show excessive use of overt markers to express definiteness, and display no preference for postverbal INTROs.

Considering the frequency effect of input observed in Paradis and Navarro (2003), we expect that the structural frequency of REs in maternal input will be reflected in bilingual children’s production.

We expect that amount of language exposure predicts bilingual children’s production of indefinite nominals for INTRO and definite/identifiable nominals for Re-INTRO in each language, given that previous findings showed a significant effect of amount of exposure in reference production in heritage speakers of Mandarin (Jia and Paradis, 2015) and in bilingual children acquiring English as an additional language (Serratrice and De Cat, 2020).

#### Cognitive factor

To what extent is Mandarin-English bilingual children’s reference production in each of the target languages influenced by working memory?

##### Predictions

Given the evidence that children with stronger working memory capacity are better able to produce felicitous REs (e.g., Torregrossa, 2017; Serratrice and De Cat, 2020), we expect that bilingual children’s working memory capacity predicts their production of indefinite nominals for INTRO and definite/identifiable nominals for Re-INTRO in Mandarin and in English.

### Participants

We recruited Mandarin-English bilingual children from a kindergarten in Singapore, where they were enrolled in a full-day Mandarin-English bilingual program, with roughly equal distribution of exposure to each language at school. Their class teachers were native speakers of either Mandarin or English and were assigned to address the children in their native language. A screening questionnaire was distributed among parents of children from classes of Nursery and Kindergarten 1 to identify families in which both Mandarin and English were spoken. 71 families met the requirement and 33 of them consented to participation. However, 12 of them did not complete the tasks. The final sample included 21 typically developing children (13 girls) between 4;5 and 6;5 (*M*age = 5;6). Parental questionnaire showed that 10 children received regular exposure to Mandarin and English from birth, and the rest started exposure to Mandarin/English from birth and English/Mandarin between 3 and 36 months. All children heard Mandarin and English from one or more caregivers and/or older siblings in the home (nine of the children had older siblings), with different amount of exposure to the two languages (see the section “Measures”). Most of the children had never lived outside Singapore for over 3 months except for one child who had visited relatives in Malaysia frequently. According to the parents’ observation, 42.9% (*n* = 9) of the children were balanced between the two languages, 38.1% (*n* = 8) were more proficient in English than in Mandarin, and the remaining 19% (*n* = 4) were more proficient in Mandarin than in English.

Mothers of the children were invited to a storytelling task performed in Mandarin and in English at the participants’ own home. Maternal input was chosen to be examined because our language exposure questionnaire data (details below) showed that the mothers were the main caregiver of their child^4^ and there are emerging research interests in the quality of input provided by bilingual mothers (e.g., Hoff et al., 2020). 81% (*n* = 17) of the mothers held Bachelor’s degrees or higher, suggesting mid to high socioeconomic status background. 57.1% (*n* = 12) and 28.6% (*n* = 6) considered themselves (near-)native in Mandarin and English, respectively. 38.1% (*n* = 8) and 42.9% (*n* = 9) rated themselves as fluent speakers of Mandarin and English, respectively. 95.2% (*n* = 20) of the mothers addressed their child in both Mandarin and English. Sixteen mothers (out of 21) completed the task in both languages. One mother who mostly spoke Mandarin to her child performed the task only in Mandarin.^5^

### Measures

We collected information on the children’s language exposure in addition to demographic information and language profiles of their main caregivers through a web-based interview with the parent(s). We measured the children’s working memory, and language proficiency in Mandarin and English, using standardized assessment tools.^6^ Participation was ascertained through parental consent forms. The study was approved by the Survey and Behavioural Research Ethics Committee of the Chinese University of Hong Kong. Descriptive statistics of background variables are given in Table 2.

**Table 2:** Descriptive statistics for the 21 child participants.

	Mean	SD	Range	IQR
*Age (months)*	66.14	6.73	53–77	10.5
*Current amount of exposure (proportion)*				
Mandarin	44.23%	0.13	26.54–78.45%	0.15
English	53.43%	0.13	21.55–73.46%	0.21
*Cumulative length of exposure (years)*				
Mandarin	2.15	0.89	0.64–4.06	1.28
English	2.21	0.99	0.69–4.13	1.87
*Working memory*^a^				
BRIEF-P (raw score)	24.2	5.25	17–35	8
BRIEF-P (t-score)	52.2	10.36	38–73	16
*Mandarin proficiency*				
MVST (raw score)	13.19	5.09	5–24	8
MVST (scaled score)	6.19	2.52	2–13	3
*English proficiency*				
PPVT (raw score)	85.14	23.51	47–125	44
PPVT (standard score)	97.71	14.69	73–126	25.5

IQR, interquartile range

a Calculated based on data of 20 children.

#### Language exposure

We used a parental questionnaire (in the form of an excel file) modeled on the BiLEC (Unsworth, 2013) to estimate the children’s relative amount of exposure to Mandarin and English concurrently and cumulatively. The parents (usually the mother) met members of the research team *via* the web conferencing software, Zoom Meetings. They answered questions about the child’s current language exposure on a weekly basis including (i) hours of interaction and language spoken with each input provider in the home and friends and relatives on average weekday and at weekends, (ii) language and hours of school and after-school activities, and (iii) language and hours of the child’s experience with media (e.g., TV, videos, books, and computer games). We calculated the proportion of time the child interacted with each input provider during waking hours and multiplied it with the percentage of Mandarin/English used by the respective input provider. The same applies to the calculation of the child’s language exposure in media/school/after-school activities. We added up the figures to derive the child’s relative amount of current exposure to Mandarin and English, respectively. For cumulative length of exposure, parents recalled (i) the frequency at which each caregiver (and school-age older siblings, if any) in the home spoke Mandarin/English for each one-year period in the child’s life, (ii) language use in daycare and/or school and/or out-of-school-care in these periods, and (iii) language use in the holidays. We averaged the frequency of Mandarin/English exposure at home for each period. We then calculated the proportion of time the child spent at home/daycare/school/out-of-school-care each year based on what is typical in Singapore and worked out the proportion of year with Mandarin/English exposure in each context. The estimates were summed up to obtain the cumulative exposure in each language. The range of current exposure in our sample is 26.5–78.4% in Mandarin (*M* = 44.2%, *SD* = 0.13), and 21.5–73.5% in English (*M* = 53.4%, *SD* = 0.13), with 16 of the bilingual children receiving a greater amount of input from English than Mandarin. Six of the children were also exposed to other languages, namely Cantonese, Hokkien, Teochew, and Japanese. Current exposure to other languages mostly accounted for less than 9% of the input except for one child (30.1%). The cumulative exposure in our sample was 0.64–4.06 years in Mandarin (*M* = 2.15, *SD* = 0.89), and 0.69–4.13 years in English (*M* = 2.21, *SD* = 0.99). Current and cumulative exposure were highly correlated in our sample for Mandarin (Pearson correlation, *r* = 0.78, *p* < 0.001) and English (*r* = 0.76, *p* < 0.001).

#### Working memory

We used the Behavior Rating Inventory of Executive Function-Preschool Version (BRIEF-P; Gioia et al., 2003) to measure WM in the preschoolers. BRIEF-P is a questionnaire completed by parents or teachers to reflect a child’s executive functions in everyday environment, using a three-point problem-oriented symptom rating scale. It has been reported to correlate to a varying degree with performance-based executive function assessment results in preschool children (e.g., Espy et al., 2011; Garon et al., 2016; O’Meagher et al., 2018). It is thus a good alternative to directly assessing the children during the pandemic. For this study, we adopted the WM sub-score of BRIEF-P as a proxy measure for children’s WM. In items relevant to WM, parents were asked to describe their child’s capacity to hold information in mind for completing a task or making a response—for instance, forgetting directions, losing track of what they are doing in the middle of an activity, unable to finish describing an event, person or story, and forgetting what they are supposed to retrieve when instructed, etc. Parents of 20 (out of 21) children completed BRIEF-P during a virtual meeting with our research assistants, approximately 6 months before the administration of the elicitation tasks and other measures.

#### Language proficiency

We used two standardized tests, namely, Peabody Picture Vocabulary Test—Fourth Edition (PPVT-4; Dunn and Dunn, 2007) and the receptive vocabulary subtest of the Taiwan version of the Wechsler Preschool and Primary Scale of Intelligence—Fourth Edition (WPPSI-IV; Wechsler, 2013) to measure language proficiency in English and Mandarin, respectively.^7^ In both tests, children were presented four colored pictures each time and their task was to select the one that matched the word they heard. PPVT-4 was administered by using digital tools from Q-global for teleassessment. WPPSI-IV Mandarin vocabulary subtest (MVST) was administered in accordance with guidelines from the test publisher for teleassessment (displaying the stimuli using a camera). A standard score between 85 and 115 on the English PPVT-4 scale and a scaled score between 7 and 12 on the MVST indicate that an examinee’s raw score is within the average of the age-matched monolinguals in the respective normative sample. It is clear from Table 2 that our bilingual children were generally more advanced in English than in Mandarin.

### Elicited narration task

Oral narratives were elicited remotely in real time with the picture sequences *Baby Birds* and *Baby Goats* from the Multilingual Assessment Instrument for Narratives (MAIN; Gagarina et al., 2012, 2015, 2019), which has been successfully used to elicit oral narratives in face-to-face settings from children speaking different languages including Mandarin (Sheng et al., 2020). The stories depict comparable character actions and emotions, and have parallel episodic structures. Both involve five characters that are familiar to young children: a mommy bird/a mommy goat, two baby birds/two baby goats, a cat/a fox, and a dog/a crow. Each story is made up of three episodes, with two pictures depicting an episode.

We adapted one of the PowerPoint templates of MAIN (Hamdani et al., 2021) for remote testing. The adaptations included the use of animation in place of videos to show the folding/unfolding of the picture sequences. The MAIN instructions were pre-recorded by two female fluent speakers of Mandarin and English respectively, following the MAIN manual (Gagarina et al., 2019; Luo et al., 2020). Each child was tested once in each language, with an interval of about 1 week between sessions. The order of language and stories was counterbalanced. Half (*n* = 10) of the children were tested in English first and Mandarin second and vice versa. Eleven children told *Baby Birds* in Mandarin and *Baby Goats* in English, and 10 told *Baby Goats* in Mandarin and *Baby Birds* in English.

**Figure 2:**
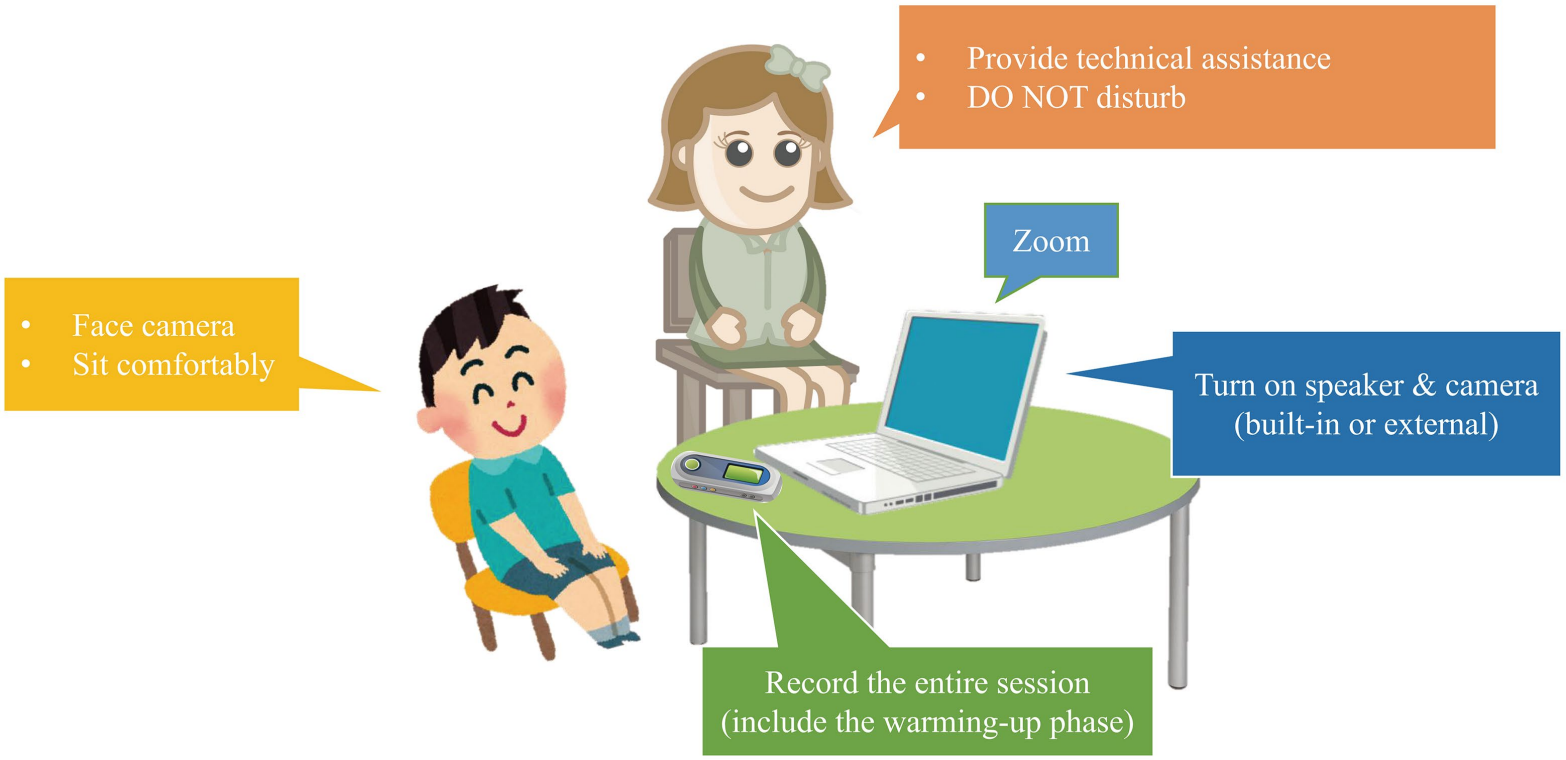
Illustration of remote elicitation of child narratives over an audio/video platform in real time.

All participants were individually tested in a quiet room at school. They were accompanied by a teacher, who provided technical assistance to the child. The teachers remained silent during the test so as not to disturb or distract the child. The child sat in front of a computer and met an experimenter based in Hong Kong *via* Zoom (illustrated in Figure 2). The experimenters (the third and fourth authors of this article) are fluent speakers of Mandarin and English but were posing as monolingual speakers of the languages, respectively, throughout the study to administer the Mandarin and English tasks separately. Test began following a short warm-up phase to establish rapport. The experimenter presented the PowerPoint using the share-screen-with-audio function of Zoom. The child viewed the shared screen in side-by-side mode, with the shared screen on the left and the video of the experimenter on the right. Three envelopes in different colors appeared on the screen and the child was asked to choose one. Whichever was chosen, the same story was presented but this was unbeknownst to the child. The child was given some time to preview the entire picture sequences. Then the pictures were “folded” and reappeared on the screen, two at a time. The child was asked to tell the story to the experimenter. Previous studies found that the presence of shared access to the referent affected children’s use of REs (e.g., Kail and Hickmann, 1992). To create the desired non-shared visual attention, the experimenters covered their eyes with their hands or a sheet of paper and made sure the child noticed it before the picture sequences were shown. The child was told to let the experimenter know when a given slide was done. By doing so, we hope to reduce the impact of screen sharing on children’s referential strategies. The session was video-recorded by the experimenter using the built-in recording function in Zoom and audio-taped by the school teacher accompanying the child using a mobile phone at the same time. The on-site audio recording was to remedy for likely unstable internet connection and subsequent loss of signals during the Zoom calls. The transcription and coding (to be introduced below) were performed based on an edited version of the Zoom video recording, in which the soundtrack was replaced by the on-site audio recording. This apparatus and setup was first created in remote web-based data collection for the Child Heritage Chinese Corpus (Mai and Yip, in prep) in CHILDES (MacWhinney, 2000) and adopted in a series of similar studies by the team (e.g., Mai et al., in prep).

### Recording home storytelling by mother

**Figure 3:**
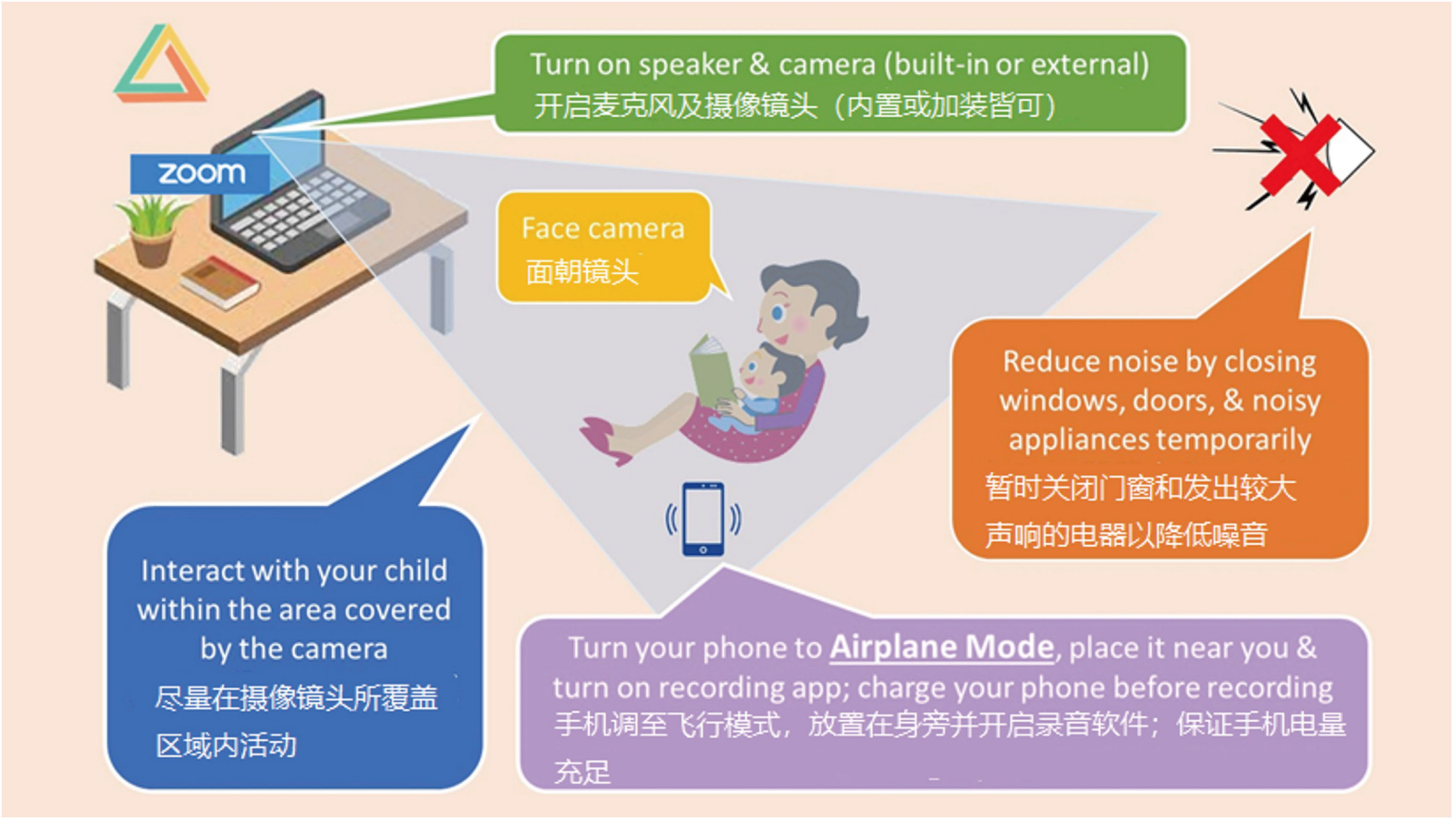
Illustration of remote recording of mother–child interactions over an audio/video platform in real time.

Participating mothers received a hardcopy of the picture sequences of the two MAIN stories (printed on A4 paper). They were asked to tell the stories to their child at home in the way they would normally do (illustrated in Figure 3). Both stories were told twice on different days, once in Mandarin and once in English. The order was determined freely by the mother. The two Hong Kong-based experimenters video-recorded the mother–child interaction with Zoom. They remained muted and invisible during the recording. Like storytelling by the children recorded in the school, additional on-site audio-recording was also obtained through the mother and edited into the video recording. It took around 5 min to complete the recording in each language.

### Transcription and coding

Children’s oral narratives and mother’s home storytelling samples were transcribed verbatim in the CHAT-format (MacWhinney, 2000) and carefully checked. Transcription included non-verbal information relevant to referential choice such as pointing during mother–child interaction, which was captured by the video recordings.

Each reference to the story characters (excluding REs used in imagined dialogues between story characters^8^) was coded in terms of referential form, syntactic position (INTRO only), and discourse function, excluding unclear or unintelligible utterances.

*Referential forms* were first coded into different RE types based on Hickmann et al. (1996) and Jia and Paradis (2015): [Num-Cl-N] (Mandarin), [Indef./Def./Num. determiner-NP] (English), bare nouns (Mandarin), no determiner singular N (used as proper nouns in English, e.g., *Cat is so naughty*), demonstrative NPs,^9^ kinship terms, complex NPs containing a possessor, a relative clause or adjectival modification, personal pronouns, demonstratives, null forms, and non-specific lexical items (e.g., *someone*).

*Syntactic position of REs in INTRO contexts* was coded as preverbal or postverbal. Cases in which position is irrelevant or cannot be determined (e.g., labeling without predication) were coded as unanalyzable and excluded from the analysis, as in Hickmann and Liang (1990).

*Discourse function* was coded largely following Serratrice (2007), with reference to Colozzo and Whitely (2014). The unit of analysis is “clause” defined by the presence of a verbal predicate (Serratrice, 2007). The verbal predicates are mainly verbs and may include adjectives in Mandarin. INTRO is the first mention of a character. Re-INTRO involves topic shift across adjacent clauses. To be coded as Re-INTRO, an RE must meet one of the following criteria: (i) a subject/object argument referring to a previously identified referent which has not been mentioned in the immediately preceding clause; (ii) a subject argument that has been mentioned in the adjacent clause as a non-subject (e.g., an object or an adjunct); or (iii) the reference shifts from two or more characters together to only one of these characters (and vice versa). The participating mothers often interacted with their child by asking questions and discussing the plots when performing the home storytelling task. Child utterances which were relevant to the thematic progress of the story were treated as part of the discourse and taken into consideration when coding the discourse functions of REs in the mother data. Examples of the coding are given in the Supplementary Material.

To assess intercoder reliability, the data were coded independently by the first author (C1) and the third and fourth authors (C3 and C2), all of whom were Mandarin-English bilinguals: C1 coded all the data, C2 coded the English child data, and C3 coded the Mandarin data and the English mother data. The agreement rate (i.e., the percentage of items with consistent coding between coders out of the total number of coded items) was 99.48% between C1 and C2 and 99.81% between C1 and C3. All inconsistencies were discussed among the coders until consensus was reached.

## Results

In total, the narratives yielded 248 REs (INTRO 82, Re-INTRO 166) in child Mandarin, 257 REs (INTRO 80, Re-INTRO 177) in child English, 777 REs (INTRO 114, Re-INTRO 663) in mother Mandarin, and 658 REs (INTRO 111, Re-INTRO 547) in mother English. For expository convenience, we further categorized the nominals into indefinite nominals and definite/identifiable nominals based on their expected interpretation in the target grammar (see Table 1). The child data were subject to Chi-square tests to rule out potential effects of story and testing order by comparing occurrences of indefinite nominals, definite/identifiable nominals, pronominals, and null forms. Results showed that the two stories elicited comparable number of REs for INTRO [χ^2^(3, *N* = 162) = 1.614, *p* = 0.656] and Re-INTRO [χ^2^(3, *N* = 343) = 2.945, *p* = 0.400], and there was no difference between English-first and Mandarin-first groups [INTRO χ^2^(3, *N* = 162) = 5.647, *p* = 0.130; Re-INTRO χ^2^(3, *N* = 343) = 0.526, *p* = 0.914].

We analyzed the distribution of REs in each participant for each language by calculating their percentages among the total number of REs for INTRO and Re-INTRO, respectively. This is to assess the similarities and differences in the use of REs between Mandarin and English, and between bilingual children’s production and maternal input. For each discourse function, we examined whether bilingual children’s production of different types of REs correlated with maternal input (in terms of structure frequency). We also implemented generalized linear mixed-effects logistic regression models to investigate the effects of linguistic, input, and cognitive factors and their interactions in bilingual reference production. Most of the statistical tests were run using IBM SPSS Statistics Version 26 except for the mixed-effects analyses, for which we used the R package *lme4* (Bates et al., 2015) in the statistical program R (version 3.5.2, R Core Team, 2018). The Shapiro–Wilk test showed that some of the variables were not normally distributed. Therefore, results of nonparametric tests will be reported unless indicated otherwise. To calculate the *post hoc* power analysis for mixed models, we employed the *simr* package (Green and MacLeod, 2016) in R. Tables 3–6 present the distribution of REs used to introduce and reintroduce characters in Mandarin and English, respectively.

**Table 3:** Descriptive statistics of the referential expressions (REs) produced by the Mandarin-English bilingual children (*n* = 21) and their mothers (*n* = 17) to introduce and reintroduce characters (INTRO, Re-INTRO) in Mandarin.

	INTRO		Re-INTRO	
	Child	Mother	Child	Mother
[Num-Cl-N]	29.27% (24)	54.39% (62)	10.84% (18)	8.6% (57)
Bare noun	18.29% (15)	9.65% (11)	15.66% (26)	38.91% (258)
Complex NP	10.98% (9)	24.56% (28)	8.43% (14)	21.87% (145)
Kinship term	2.44% (2)	0	1.2% (2)	6.64% (44)
Demonstrative NP	35.37% (29)	4.39% (5)	48.19% (80)	10.26% (68)
Demonstrative	0	7.02% (8)	0	1.21% (8)
Personal pronoun	2.44% (2)	0	12.65% (21)	8.14% (54)
Null form	1.22% (1)	0	3.01% (5)	4.37% (29)

**Table 4:** Pre/postverbal positioning of referential expressions (REs) in INTROs in Mandarin-English bilingual children and their mothers: Mandarin.

	Child (*n* = 21)		Mother (*n* = 17)	
	Preverbal	Postverbal	Preverbal	Postverbal
Overall	71.95% (59)	28.05% (23)	44.44% (48)	55.56% (60)
[Num-Cl-N]	45.83% (11)	54.17% (13)	8.62% (5)	91.38% (53)
Bare nouns	86.67% (13)	13.33% (2)	90.9% (10)	9.09% (1)
Complex NP	66.67% (6)	33.33% (3)	76.92% (20)	23.08% (6)
Kinship term	50% (1)	50% (1)	0	0
Demonstrative NP	93.1% (27)	6.9% (2)	100% (5)	0
Demonstrative	0	0	100% (8)	0
Personal pronoun	50% (1)	50% (1)	0	0
Null form	0	100% (1)	0	0

**Table 5:** Descriptive statistics of the referential expressions (REs) produced by the Mandarin-English bilingual children (*n* = 21) and their mothers (*n* = 16) to introduce and reintroduce characters (INTRO, Re-INTRO) in English.

	INTRO		Re-INTRO	
	Child	Mother	Child	Mother
[indef. determiner-NP]	21.25% (17)	36.04% (40)	0	2.19% (12)
[num. determiner-NP]	11.25% (9)	16.22% (18)	4.52% (8)	3.11% (17)
[def. determiner-NP]	62.5% (50)	26.13% (29)	85.31% (151)	70.38% (385)
No determiner singular N	1.25% (1)	0.9% (1)	0.56% (1)	0.37% (2)
Complex NP	2.5% (2)	9.91% (11)	2.26% (4)	10.05% (55)
Kinship term	0	0	0	1.65% (9)
Demonstrative NP	1.25% (1)	5.41% (6)	0.56% (1)	2.38% (13)
Demonstrative	0	2.7% (3)	0	0.91% (5)
Personal pronoun	0	0.9% (1)	4.52% (8)	7.5% (41)
Null form	0	0	2.26% (4)	1.46% (8)
Non-specific lexical item	0	1.8% (2)	0	0

**Table 6:** Pre/postverbal positioning of referential expressions (REs) in INTROs in Mandarin-English bilingual children and their mothers: English.

	Child (*n* = 21)		Mother (*n* = 16)	
	Preverbal	Postverbal	Preverbal	Postverbal
Overall	68.42% (52)	31.58% (24)	48.91% (45)	51.09% (47)
[indef. determiner-NP]	41.18% (7)	58.82% (10)	37.84% (14)	62.16% (23)
[num. determiner-NP]	44.44% (4)	55.56% (5)	21.43% (3)	78.57% (11)
[def. determiner-NP]	85.11% (40)	14.89% (7)	68.18% (15)	31.82% (7)
No determiner singular N	0	0	100% (1)	0
Complex NP	0	100% (2)	71.43% (5)	28.57% (2)
Demonstrative NP	100% (1)	0	80% (4)	20% (1)
Demonstrative	0	0	33.33% (1)	66.67% (2)
Personal pronoun	0	0	100% (1)	0
Null form	0	0	0	0
Nonspecific lexical item	0	0	50% (1)	50% (1)

### Referential choice for introduction of characters

#### Mandarin

Compared to the mothers, the bilingual children produced more demonstrative NPs (35.37% vs. 4.39%; Mann–Whitney test, *U* = 84, *z* = −3.01, *p* = 0.003), fewer [Num-Cl-N] (29.27% vs. 54.39%; *U* = 87, *z* = −2.706, *p* = 0.007), and a lower rate of complex NPs (10.98% vs. 24.56%; *U* = 97, *z* = −2.051, *p* = 0.012) for INTRO in Mandarin. They produced more bare nouns (18.29% vs. 9.65%) than the mothers, though the difference was non-significant (*p* = 0.484). Pronominals and null forms were used infrequently (0–7.02%) for INTRO by the children and the mothers. We performed bivariate correlation tests to find out the relations between children’s production and maternal input in terms of structural frequency. A significant correlation was found for the use of bare nouns in Mandarin INTRO contexts (Spearman’s rank correlation, *r_s_* = 0.482, *p* = 0.05).

The INTROs in child Mandarin were more often preverbal than postverbal (71.95% vs. 28.05%) (Wilcoxon signed-rank tests, *z* = −2.541, *p* = 0.011). Pre- and postverbal INTROs (44.44% vs. 55.56%) were almost equally distributed in the maternal input (*p* = 0.477). The children produced significantly fewer postverbal INTROs than the mothers (*U* = 95, *z* = −2.45, *p* = 0.014).

About half of the [Num-Cl-N] were preverbal in the bilingual children (54.17%), whereas most [Num-Cl-N] appeared postverbally in the maternal input (91.38%). Bare nouns were mostly preverbal (child 86.67%, mother 90.9%); so were demonstrative NPs (child 93.1%, mother 100%).

#### English

For INTRO in English, our bilingual children differed from the mothers in producing more [Def. determiner-NP] (62.5% vs. 26.13%; *U* = 62, *z* = −3.272, *p* = 0.001), fewer [Indef. Determiner-NP] (21.25% vs. 36.04%; *U* = 100, *z* = −2.136, *p* = 0.033) and a lower rate of complex NPs (2.5% vs. 9.91%; *U* = 104, *z* = −2.510, *p* = 0.012). While [Num. determiner-NP] was used occasionally (child 11.25%, mother 16.22%), the use of the other RE types was rare (child 0–1.25%, mother 0–5.41%). There was no significant mother–child correlation regarding structural frequency of REs in English INTRO contexts (*p*s > 0.4).

The INTROs in English were mostly preverbal as opposed to postverbal (68.42% vs. 31.58%) in the bilingual children (*z* = −2.583, *p* = 0.01) and almost equally distributed between the pre- and postverbal positions in the mothers (48.91% vs. 51.09%, *p* = 1). The children produced significantly fewer postverbal INTROs than the mothers (*U* = 96.5, *z* = −2.212, *p* = 0.027).

[Indef. determiner-NP] and [Num. determiner-NP] were often postverbal in the bilingual children (Indef. 58.82%, Num. 55.56%), and mostly postverbal in the mothers (Indef. 62.16%, Num. 78.57%). By contrast, most [Def. determiner-NP] appeared preverbally (child 85.11%, mother 68.18%).

### Referential choice for re-introduction of characters

#### Mandarin

For Re-INTRO in Mandarin, demonstrative NPs were used most frequently by the bilingual children (48.19%), followed by bare nouns (15.66%), personal pronouns (12.65%), and complex NPs (8.43%). This contrasts with the maternal input, in which bare nouns (38.91%) and complex NPs (21.87%) were used more frequently than demonstrative NPs (10.26%) and personal pronouns (8.14%). The child–mother differences were significant with demonstrative NPs (*U* = 83.5, *z* = −2.804, *p* = 0.005), bare nouns (*U* = 60, *z* = −3.551, *p* < 0.001), and complex NPs (*U* = 47, *z* = −3.924, *p* < 0.001). [Num-Cl-N] was used occasionally by the children (10.84%) and the mothers (8.6%). The use of demonstratives and null forms for Re-INTRO was infrequent in Mandarin (0–4.37%). A positive mother–child correlation was found with the frequency of demonstrative NPs in Mandarin Re-INTRO contexts (*r_s_* = 0.548, *p* = 0.023). The bilingual children’s use of demonstrative NPs increased as the frequency of demonstrative NPs in the maternal input increased.

#### English

Our children’s Re-INTROs in English were patterned after the maternal input: [Def. determiner-NP] occurred the most frequently (child 85.31%, mother 70.38%), while the other REs were infrequent (child 0–4.52%, mother 0–7.5%), except that the mothers showed occasional use of complex NPs (10.05%). There was no significant mother–child correlation regarding structural frequency of specific types of REs in English Re-INTRO contexts (*p*s > 0.2).

### Multifactorial modeling

We generated four mixed-effects logistic regression models. Two modeled the bilingual children’s reference production in Mandarin (Model 1) and English (Model 2), and the others modeled their reference production for INTRO (Model 3) and Re-INTRO (Model 4). In these models, the referential choice was entered as binary data and participants were treated as a random effect.^10^ Categorical factors were sum-coded (i.e., −0.5 and 0.5) and continuous variables were mean centered (by subtracting the mean from the value). Since a higher score obtained in the BRIEF-P assessment suggests weaker executive functions, the working memory scores were reversed by multiplying “-1” after the mean-centering procedure to align with other variables. Two-way interactions between predictor variables were included if they significantly improve model fit as measured by Akaike Information Criterion.

For Models 1 and 2, the dependent variable was the choice between definite/identifiable nominals and others. As fixed effects, we entered (i) Discourse function as a two-level factor (INTRO, Re-INTRO), (ii) continuous predictors including Cumulative length of exposure^11^ in Mandarin/English, Working memory (raw scores), and language proficiency (MVST/PPVT raw scores). The interaction between discourse function and English language proficiency was included in Model 2 as it significantly improved model fit. Tables 7, 8 show the results.

**Table 7:** Results from a mixed-effects logistic regression model on Mandarin-English bilingual children’s (*n* = 20) choice of definite nominals vs. other REs in the Mandarin oral narrative task (229 observations).

Predictor	Estimate	SE	*z* value	*p* value
Intercept	0.97	0.199	4.868	< 0.001^***^
Discourse function (Re-INTRO vs. INTRO)	0.134	0.163	0.825	0.409
Cumulative length of exposure (Mandarin)	−0.101	0.238	−0.425	0.671
Working memory	0.024	0.043	0.558	0.577
Mandarin proficiency (MVST raw scores)	0.026	0.044	0.585	0.559

*** *p* < 0.001.

**Table 8:** Results from a mixed-effects logistic regression model on Mandarin-English bilingual children’s (*n* = 20) choice of definite nominals vs. other REs in the English oral narrative task (243 observations).

Predictor	Estimate	SE	*z* value	*p* value
Intercept	1.956	0.298	6.568	< 0.001^***^
Discourse function (Re-INTRO vs. INTRO)	0.412	0.251	1.639	0.101
Cumulative length of exposure (English)	−0.261	0.326	−0.8	0.424
Working memory	0.011	0.053	0.213	0.831
English proficiency (PPVT raw scores)	−0.041	0.016	−2.639	0.008^**^
Discourse function × English proficiency	0.028	0.012	2.301	0.021^*^

*** *p* < 0.001;

** *p* < 0.01;

* *p* < 0.05.

#### Mandarin (Model 1)

No significant effect was found (*p*s > 0.4).

#### English (Model 2)

There was a significant main effect of language proficiency (*β* = −0.041, *SE* = 0.016, *z* = −2.639, *p* = 0.008; *post hoc* power = 84.4%), which was qualified by discourse function (*β* = 0.028, *SE* = 0.012, *z* = 2.301, *p* = 0.021; *post hoc* power = 94.7%). That is, the production of definite/identifiable nominals (infelicitous) in INTRO contexts decreased as proficiency increased, while the probability of definite/identifiable nominals (felicitous) in Re-INTRO contexts was similarly high across proficiency. No effect of cumulative exposure to English was found (*p* = 0.424).

For Models 3 and 4, the dependent variable was indefinite nominals vs. others and definite/identifiable nominals vs. others, respectively. As fixed effects, we entered (i) Language as a two-level factor (English, Mandarin), (ii) continuous predictors including Structural frequency (of indefinite nominals for INTRO or definite/identifiable nominals for Re-INTRO) in the maternal input, Working memory (raw scores), Relative cumulative exposure (subtracting the child’s cumulative length of exposure to Mandarin from her/his cumulative length of exposure to English), and Age. The interaction between structural frequency and working memory was included in both models as it significantly improved model fit. Tables 9, 10 show the results.

**Table 9:** Results from a mixed-effects logistic regression model on Mandarin-English bilingual children’s (*n* = 17) choice of indefinite nominals vs. other REs in INTRO contexts (128 observations).

Predictor	Estimate	SE	*z* value	*p* value
Intercept	−1.027	0.268	−3.836	<0.001^***^
Language (Mandarin vs. English)	−0.209	0.219	−0.958	0.338
Structural Frequency	−1.519	1.1	−1.382	0.167
Working memory	0.06	0.063	0.953	0.341
Relative cumulative length of exposure	1.29	1.213	1.064	0.287
Age	0.083	0.046	1.814	0.07
Structural frequency × Working memory	0.674	0.22	3.064	0.002^**^

*** *p* < 0.001;

** *p* < 0.01.

Structural frequency, frequency of indefinite nominals in the maternal input; Relative cumulative length of exposure, English-Mandarin differences in cumulative length of exposure.

**Table 10:** Results from a mixed-effects logistic regression model on Mandarin-English bilingual children’s (*n* = 17) choice of definite/identifiable nominals vs. other REs in Re-INTRO contexts (289 observations).

Predictor	Estimate	SE	z value	p value
Intercept	1.819	0.268	6.777	<0.001^***^
Language (Mandarin vs. English)	−0.694	0.198	−3.508	<0.001^***^
Structural frequency	5.323	2.921	1.822	0.068
Working memory	0.031	0.053	0.584	0.559
Relative cumulative length of exposure	−0.567	1.082	−0.524	0.6
Age	0.019	0.04	0.485	0.628
Structural frequency × Working memory	1.224	0.616	1.986	0.047^*^

*** *p* < 0.001;

* *p* < 0.05.

Structural frequency, frequency of definite/identifiable nominals in the maternal input; Relative cumulative length of exposure, English-Mandarin differences in cumulative length of exposure.

#### INTRO (Model 3)

There was a marginally significant age effect (*β* = 0.083, *SE* = 0.046, *z* = 1.814, *p* = 0.07; *post hoc* power = 49%), suggesting a trend in more indefinite nominals (felicitous for INTRO) with increasing age. Working memory interacted with structural frequency (*β* = 0.674, *SE* = 0.220, *z* = 3.064, *p* = 0.002; *post hoc* power = 98.2%): children with stronger working memory capacity and higher frequency of indefinite nominals in the maternal input were more likely to produce indefinite nominals in introducing characters. Whether the children received more exposure to English than to Mandarin did not show any significant effects on the production of indefinite nominals in INTRO contexts (*p* = 0.287).

#### Re-INTRO (Model 4)

Language was a significant predictor (*β* = −0.694, *SE* = 0.198, *z* = −3.508, *p* < 0.001; *post hoc* power = 96.7%). The bilingual children produced more definite/identifiable nominals (felicitous for Re-INTRO) in English than in Mandarin. Structural frequency showed a marginally significant main effect (*β* = 5.323, *SE* = 2.921, *z* = 1.822, *p* = 0.0068; *post hoc* power = 59.5%), and interacted with working memory (*β* = 1.224, *SE* = 0.616, *z* = 1.986, *p* = 0.047; *post hoc* power = 56.7%). In other words, children with stronger working memory capacity produced more definite/identifiable nominals for Re-INTRO with increasing frequency of these nominals in the maternal input. The production of definite/identifiable nominals in Re-INTRO contexts did not change as a function of relative cumulative exposure (*p* = 0.6).

## Discussion

### Summary of main findings

The current study investigated the relationship between reference production on the one hand, and linguistic, input, and working memory on the other by examining referential choice in 4–6-year-old Singaporean Mandarin-English bilingual children through a bilingual elicited narration task, supplemented by a battery of language proficiency, input and cognitive measures.

Our first research question concerns bilingual children’s referential choice for INTRO and Re-INTRO contexts. The results showed that our bilingual children overused definite nominals for INTRO in both Mandarin and English. The use of indefinite nominals in INTRO contexts improved as a function of language proficiency with English but not with Mandarin. Although we did not include monolingual groups in this study, below we make comparisons drawing on the trends and patterns in the English/Mandarin monolingual preschoolers reported in Hickmann et al. (1996) (hereafter HHRL) and 5-year-old Mandarin monolinguals in Wu et al. (2015) (hereafter WHZ) in terms of INTRO contexts. Both HHRL and WHZ tested children’s reference production using elicited narration tasks similar to our study.

In English, our bilingual children produced more [Def. determiner-NP] (62.5%) than [Indef. determiner-NP] (21.25%), similar to the English monolingual peers (HHRL 62% vs. 25%). Different patterns of results were observed in Mandarin, however. Our bilingual children produced more demonstrative NPs (35.37%) than [Num-Cl-N] (29.27%) and bare nouns (18.29%), while Mandarin monolingual preschoolers used [Num-Cl-N] (HHRL 50%, WHZ 47–73%) and bare nouns (HHRL 34%, WHZ 27–49%) more frequently than demonstrative NPs (HHRL 17%, WHZ 0–4%). First mentions were more often preverbal than postverbal in both our bilingual children (68.42% vs. 31.58%) and the English monolingual children (HHRL around 70% vs. 30%). The same holds in Mandarin, though the difference in proportions seems to be larger in our bilingual children (72% vs. 28%) than in the monolingual children (HHRL 56% vs. 44%, WHZ 64.68% vs. 35.32%). This is partially consistent with Jia and Paradis (2015), who reported no preference for the postverbal position in heritage Mandarin speakers, despite that first mentions are typically postverbal in Mandarin.

Re-INTRO constitutes felicitous contexts for definite/identifiable nominals. As expected, definite/identifiable nominals were more frequent in Re-INTRO contexts than INTRO contexts, especially in English, as revealed by the mixed-effects analysis. The higher rate of definite/identifiable nominals in English than in Mandarin is expected and probably attributable to Mandarin-English differences independent of bilingualism, as the same pattern was found in the monolingual children (Mandarin 69.2%, English 84.4%) in Chen and Lei (2012). Our bilingual children produced a higher frequency of demonstrative NPs (80 out of 140 nominals, 57.14%) than the 6–9-year-old typically developing Mandarin monolingual children in Sah (2018) (24 out of 276 nominals, 8.7%) for Re-INTRO. This echoed the bilingual-monolingual differences in demonstrative use between heritage Mandarin speakers and homeland speakers reported in Aalberse et al. (2017) and Mai et al. (2021).

The results overall show that our bilingual children were sensitive to differential uses of REs in INTRO and Re-INTRO contexts while overproducing definite nominals for INTRO. Meanwhile, they showed specific uses of REs in Mandarin (partially) consistent with previous findings, including an increase in the use of demonstrative NPs and the prevalence of preverbal INTROs, which will be returned to in the next section.

Our second research question examines differences and correlation between children’s reference production and maternal input in terms of structural frequency, and the role of input in bilingual reference production. We performed a qualitative analysis of the REs in the children and their mothers. The referential choice in our bilingual children generally patterned with that by their mothers except for two child–mother differences:^12^ the children (i) produced indefinite nominals less frequently and preferred the preverbal position in INTRO contexts, and (ii) employed overt marking to code definiteness more frequently in Mandarin. Correlation analyses revealed positive relations between the children and the mothers in terms of structural frequency of (i) bare nouns for INTRO in Mandarin, and (ii) demonstrative NPs for Re-INTRO in Mandarin. Mixed-effects analyses showed that the frequency of felicitous REs produced by the children increased with a higher frequency of felicitous REs in the maternal input, modulated by working memory in both INTRO and Re-INTRO contexts. These results are consistent with the observation in Paradis and Navarro (2003), suggesting that our bilingual children were sensitive to the structural frequency in the input, which impacted on the patterns of their reference use. Amount of language exposure turned out to show no predicting effect on reference production, which is inconsistent with previous studies (e.g., Jia and Paradis, 2015). We will return to this in the section “Role of input in bilingual reference production.”

Our third research question investigates the effect of working memory on bilingual reference production. As mentioned, there was a modulating effect of working memory on the mother–child association in the production of felicitous REs regardless of discourse context and language. Children with stronger working memory capacity and more frequent felicitous REs in the maternal input were better able to produce felicitous REs. These results are in line with previous evidence of working memory influencing child reference production (e.g., Torregrossa, 2017; Serratrice and De Cat, 2020).

### Specific uses of REs in bilingual reference production

Compared to maternal input, our bilingual children under-produced indefinite nominals in INTRO contexts in English and Mandarin as expected. As proficiency increased, they produced a higher frequency of indefinite nominals for INTRO in English but not in Mandarin. This suggests that linguistic properties involving information structure and discourse such as REs in Mandarin could be particularly vulnerable in bilingual grammars, consistent with existing patterns in other bilingual populations (e.g., Mai and Deng, 2019).

Our children showed non-adult-like preference for the preverbal position when mentioning new referents. This is shown in (7), in which a more appropriate structure in Mandarin is SV inversion (i.e., *ranhou lai le yi-ge huli* “then came a fox”).

7. Ranhou yi-ge huli lai le.

then one-cl fox come le

Intended: “Then a fox came.” (JL, 5;11)

One explanation for the “preverbal” preference is young children’s preference of novelty. While the clause-initial position is typically associated with highly accessible referents (e.g., already mentioned, hence activated, and accessible) in adult speech, it may also be associated with novelty and change, resulting in new information being mentioned first (Bock et al., 2004). It has been shown that young children organize their sentences prioritizing novelty rather than accessibility, preferring to highlight new information first (Chen and Narasimhan, 2018; Chen et al., 2020). The preverbal preference observed in our study is consistent with these studies. Another possibility may lie in the differences in focus-marking between the two languages in general: Mandarin relies heavily on word order and syntactic constructions for focus-marking, whereas in English focus has a systematic manifestation *via* pitch accent, with less reliance on word order variation for realization (Chen et al., 2016). We conjecture that sustained exposure to English might have weakened the association between newness and postverbal positions in bilingual Mandarin grammars. The extent to which the preverbal preference is influenced by focus-marking of English needs further investigation.

Our expectation that bilingual children would show a high frequency of demonstrative NPs is confirmed. As shown in (8–9), our bilingual children frequently used the demonstrative *na* “that” plus a classifier (e.g., *na-ge*) to overtly mark definiteness, which is semantically redundant in Mandarin but well explained if *na* “that” was reanalyzed as the definite article *the* in English.

8. Ranhou na-ge gou yao zhua na-ge mao.

then that-cl dog want catch that-cl cat

“Then the dog wanted to catch the cat.” (GX, 5;7)

9. Na-ge hei niao zhui zhe na-ge huli.

that-cl black bird chase asp that-cl fox

“The black bird was chasing the fox.” (LJ, 5;11)

Demonstratives in Mandarin are akin to the definite article in English in situations such as noncontrastive anaphoric reference and restrictive relative clauses (Chen, 2004). The obligatory use of the definite article in English might have trigged the search for an equivalent morpheme in Mandarin. Another possibility may be related to tolerance of “redundancy” due to a general effect of bilingualism—for example, the need to deal with higher processing load (Sorace and Filiaci, 2006; Sorace et al., 2009). It has been observed that bilingual children tend to be “over-explicit” in reference production, regardless of language combinations. They overused overt subjects/objects in contexts where a null form or clitic would be more appropriate (Paradis and Navarro, 2003; Belletti et al., 2007), and produced full nouns under circumstances where the use of pronominals is expected (Torregrossa et al., 2021). It follows that cross-linguistic influence and a general effect of bilingualism may jointly underlie the increase in the production of demonstrative NPs as observed in our data. Further research is needed to distinguish between the causes by comparing the use of demonstrative NPs in bilingual children acquiring two article-less languages. If those children also show over-reliance on overt marking of definiteness as our children did, it is likely due to a general effect of bilingualism.

### Role of input in bilingual reference production

How input shapes language development is a question that features prominently in language-acquisition research. Recent studies have addressed this question by using various measures of linguistic input to predict children’s language proficiency (e.g., Place and Hoff, 2016) or by investigating differential relations between input and acquisition of different linguistic structures (e.g., Paradis et al., 2014; Unsworth, 2014). The present study adds to the existing literature by presenting additional evidence regarding effects of different aspects of input in dual-language environments on bilingual reference production.

Our results highlight the robust correlation between structural frequency of REs in the input and patterns of reference use in 4–6-year-old Mandarin-English bilingual children. Maternal input positively correlated with children’s production of specific types of REs, and importantly, structural frequency in the input interacted with working memory in predicting the bilingual children’s production of felicitous REs (indefinite nominals for INTRO and definite/identifiable nominals for Re-INTRO) across languages. Thus, the results provide further support for Paradis and Navarro’s (2003) observation that structural frequency in the input may be another source contributing to variation in children’s referential choice. Note that REs produced by the children and the parents were collected and measured separately in different recording sessions. Although the mother told the stories in the presence of the child at home, the child told the stories in the kindergarten without the mother. This effectively reduces the possibility that mother–child associations in RE production are merely temporary adaptation effects between conversation interlocutors in general. Rather, the associations truly reflect the role of input on the acquisition outcomes in a longer term.

Recall that the production of indefinite nominals in Mandarin INTRO contexts is particularly challenging for our bilingual children. The input that the children received played a role here. As suggested by the structural frequency effect that was modulated by working memory, children who heard a higher frequency of indefinite nominals in the input and had stronger working memory capacity were better able to produce them. Following this, insufficient cues instantiating felicitous REs in the input may hamper children’s development of referential abilities. Take bare nouns for example. It turned out that the mothers seldom produced bare nouns in INTRO contexts (9.65%) and when they did, most of the bare nouns they produced were preverbal (90.9%). The predominance of preverbal bare nouns over postverbal ones in INTRO contexts is unexpected since the opposite is believed to be the norm in Mandarin. Interestingly, in Wu et al. (2015), the adult controls who were university students speaking the homeland variety of Mandarin as the native language also produced up to 41% of bare nouns in the preverbal position in INTRO contexts. The empirical evidence from the current study and Wu et al. (2015) both point toward a less significant tendency for bare nouns to appear postverbal in reference to new entities in Mandarin, compared to what was described in the theoretical literature (e.g., Cheng and Sybesma, 1999). Whether this discrepancy can be explained by contact-induced variation and change in Mandarin [e.g., contact influence from English which does not employ word order to mark (in)definiteness] awaits further investigation. In either case, ambiguity naturally arises in the input regarding the interpretation of bare nouns in pre- and postverbal positions from the perspective of acquisition. The input could be even less robust in our bilingual children than that of monolinguals, as the relevant amount of data in the input is reduced relative to monolingual children. Under such circumstances, it would be difficult for bilingual children to associate postverbal bare nouns with indefiniteness and preverbal ones with definiteness. This was borne out in our study, with most of the bare nouns for INTRO (86.67%) being preverbal in our children. Thus, our study suggests that less robust input with insufficient frequency of relevant structures would render REs more vulnerable for acquisition.

For now, we found no significant main effects of amount of language exposure on bilingual reference production. This appears to contradict the finding of Jia and Paradis (2015) who reported a significant effect of age of arrival on first mention abilities in 6–10-year-old heritage Mandarin children in Canada. It should be noted, however, that their children arrived at Canada at a rather young age (24 months on average) and developed bilingualism in one context-one language environment. Importantly, the effect of age of arrival was only observed in children who attended English-only schools (HL-ENG group), rather than in those who attended Mandarin-English bilingual public schools (HL-BIL group). In the current study, our bilingual children had been living in Singapore since birth, receiving exposure to both languages in diverse contexts. They were attending a Mandarin-English bilingual program in kindergarten with relatively balanced distribution of exposure between the two languages. Given this, the null effect of amount of language exposure in the current study (and perhaps the HL-BIL group in Jia and Paradis (2015)) may well result from the threshold effect of language exposure and the potentially non-linear nature between language exposure and language outcome (Pearson, 2007; Thordardottir, 2014). That is, the amount of input that our bilingual children received might have passed a certain amount above which increases in exposure would not add to performance in reference production.

The above said, it could be that the true effect of amount of language exposure in bilingual reference production will only emerge in a more focused design with stronger statistical power and more sensitive experimental tools. Following up on our behavioral findings, future research may further assess the effect of amount of exposure to evaluate this possibility by investigating a larger sample of children with a wide array of language dominance profiles.

### Role of cognitive skills in bilingual reference production

In our study, working memory did not appear to make a significant individual contribution. Nevertheless, our results suggest that strong working memory capacity is particularly beneficial for RE acquisition among children who received input containing a higher frequency of felicitous nominals. This is consistent with studies that showed individuals with better working memory abilities are more efficient in attending to and decoding various features in the input (e.g., Sunderman and Kroll, 2009; Indrarathne and Kormos, 2018). Better working memory may assist in keeping information active for further processing and retaining it in the long-term memory, which expedites the retrieval of representations and extend the scope of attention (Martini et al., 2015), but this happens on the condition that there are sufficiently frequent cues in the input for the child to process.

The modulating effects of working memory are also in line with suggestions that better working memory helps bilingual children store, monitor, and update the addressee’s perspective in their mind (De Cat, 2015; Serratrice and De Cat, 2020). As mentioned in the section “Working memory and reference production,” reference to characters in storytelling tax working memory. Referential choice for INTRO is guided by the speaker’s presupposition about the listener’s knowledge, and Re-INTRO requires monitoring not only the knowledge state but also the attentional state of the listener (whether the character of concern is the attentional focus of the listener) to keep track of characters who are moving in and out of the attentional foreground and update the discourse model accordingly. In this sense, our result also aligns with previous research in which cognitive effects are shown to be pronounced in more complex working memory tasks (e.g., Morales et al., 2013; Blom et al., 2014).

Practical implications of the modulating effects of working memory on the mother–child association in reference production are two-fold: In multi-factorial predictive models of bilingual acquisition, pinpointing the role of working memory and its interaction with linguistic and input factors in RE acquisition facilitates more accurate predictions and expectations on the language developmental outcomes in bilingual children, given that RE is a prominent and challenging aspect in language. On the other hand, in intervention programs for bilingual children, pedagogical and educational effort can be made to utilize the positive and potentially reciprocal relation between working memory and language learning in order to promote mutual benefits for both sides. Several studies have shown that after training and intervention, working memory can improve and enhance language learning in children (see review in Archibald, 2017). In the opposite direction, there is also evidence of significant improvements in working memory after intervention targeting language skills such as phonological awareness skills (van Kleeck et al., 2006), vocabulary, and morphosyntax (Ebert, 2014) in preschool and school-age children with specific language impairment.

## Conclusion

This study investigated 4–6-year-old Mandarin-English bilingual children’s reference production, and its relationship with linguistic, input, and cognitive factors. It is the first study of narrative production that has included transcript-based analysis of the maternal input available to preschool bilingual children captured through mother–child interactions. The current study is also one of the few studies that have elicited child and adult discourse remotely online using videoconference-based methods supplemented by on-site audio-recording.

Our data showed prolonged development of indefinite nominals to introduce a new referent (INTRO) in both languages of our bilingual children, who also demonstrated over-reliance on overt-marking of definiteness in Mandarin. The results corroborate previous studies on children’s referential abilities, suggesting that linguistic properties involving morphosyntactic structure, information structure, and discourse could be particularly vulnerable in bilingual grammars. Regarding the role of input, our results underscore the importance of structural frequency in the input in shaping patterns of bilingual reference production. We have discovered mother–child association in the production of felicitous REs, the strength of which was modulated by working memory across language and discourse function. Amount of exposure did not seem to predict referential choice in our bilingual children. We postulated that there might be thresholds for amount of exposure to influence reference production. These findings shed lights on how language, input and cognitive skills might jointly influence bilingual reference production. They have direct relevance and precise implications for practice. To boost the acquisition of REs, which involve syntax-semantics-discourse interfaces, increasing the amount of input would not work best for bilingual children with relatively high proficiency in both languages, and bilingual children with different working memory capacities may benefit from different pedagogical strategies tailored for them. Those equipped with better working memory may display immediate benefit from increased frequency of REs in the input, and those with weaker working memory may need supplementary training on working memory to show similar progress, presumably not only in RE but in language learning in general.

The findings of this small-scale exploratory study await replications with a larger sample of children with different language combinations and an array of language dominance profiles. Further investigations may tease apart cross-linguistic influence effect and study the threshold effect of amount of language exposure with a more focused design using multiple linguistic and cognitive measures (e.g., combining both performance-based tests and caregiver ratings).

## Data availability statement

The raw data supporting the conclusions of this article will be made available by the authors, without undue reservation.

## Ethics statement

The studies involving human participants were reviewed and approved by the Survey and Behavioural Research Ethics Committee of the Chinese University of Hong Kong. Written informed consent to participate in this study was provided by the participants' legal guardian/next of kin.

## Author contributions

VY, ZM, and JZ designed the study and established collaboration with the kindergarten. JZ prepared the experimental materials and procedures, and revised and improved on the Zoom-based data collection protocols provided by ZM, with QC and YL’s assistance. JZ, QC, and YL recruited the participants and collected the data. QC and YL transcribed and coded most of the data under the supervision of JZ. JZ analyzed and interpreted the data in consultation with ZM and VY. JZ wrote the first and second drafts of the paper. ZM revised both. All authors worked on refining the text. All authors contributed to the article and approved the submitted version.

## Funding

We would like to acknowledge funding support from the project “Development of Bilingualism in Chinese-speaking and Overseas Communities” conducted by our three labs: the University of Cambridge – Chinese University of Hong Kong Joint Laboratory for Bilingualism, the Chinese University of Hong Kong – Peking University – University System of Taiwan Joint Research Centre for Language and Human Complexity, and the Childhood Bilingualism Research Centre at The Chinese University of Hong Kong. The work described in this paper was also partially supported by a General Research Fund from the Research Grants Council of the Hong Kong Special Administrative Region, China (Project No. CityU 14615820), awarded to ZM.

## Conflict of interest

The authors declare that the research was conducted in the absence of any commercial or financial relationships that could be construed as a potential conflict of interest.

## Publisher’s note

All claims expressed in this article are solely those of the authors and do not necessarily represent those of their affiliated organizations, or those of the publisher, the editors and the reviewers. Any product that may be evaluated in this article, or claim that may be made by its manufacturer, is not guaranteed or endorsed by the publisher.

## Footnotes

^1^Mandarin allows the use of null forms for referents that are readily identifiable in the immediately preceding discourse or physical context. However, seemingly appropriate null forms in Re-INTRO contexts does not necessarily reflect good discourse integration ability in the speaker because the appropriateness of null forms may reflect the minds of the listener more than the minds of the speaker.

^2^If a bare noun follows a verb denoting unbounded states, e.g., *xihuan* ‘like’, it receives a generic reading (Sybesma, 1992).

^3^Collection of child-directed speech from those children’s teachers at the school (i.e., teacher input) is in preparation.

^4^The proportions of maternal input in total language input were 4–24% in Mandarin and 1–16% in English.

^5^It was the older sibling (10-year-old) who communicated with the child in English at home. The remaining 4 mothers were not available.

^6^We also collected other individual difference measures which are not the focus of the current study and therefore not reported here.

^7^Although PPVT-4 and WPPSI-IV were normed on native speakers of English in the U.S and native speakers of Mandarin Chinese in Taiwan, respectively, they are a pragmatic solution that can provide independent measures of our bilingual children’s proficiency in English and Mandarin, given the lack of culturally appropriate/neutral standard tests that target bilingual children. Recall that the primary interest of the current study is not bilingual versus monolingual differences in absolute terms but relationships between variables within the bilingual group.

^8^The REs in imagined dialogue reflected the perspective of story characters rather than the perspective of the narrator or the listener.

^9^When coding the Mandarin data, we excluded cases in which the demonstrative *nage* could be treated as discourse gap fillers (i.e., there was a long pause between the demonstrative and the nominal).

^10^Only random intercepts were included. Models with random slopes either failed to converge or were not a better fit of the data as indicated by anova() comparisons. The patterns of results did not change in fuller models.

^11^The same pattern of results was obtained when current exposure was entered. This holds for the other mixed analyses. We therefore modelled the data with cumulative exposure throughout the paper for consistency.>

^12^Our mothers produced fewer [Num-Cl-N] (54.39%) for INTRO in Mandarin than the adult controls in HHRL (81%) and WHZ (79–94%). Likewise, they produced fewer [Indef. determiner-NP] (36.04% vs. 76%) and more [Def. determiner-NP] (26.13% vs. 9%) for INTRO in English than the adults in HHRL. This is because our mothers told the stories during shared reading activities with their child (we deliberately chose shared reading to capture natural mother–child interaction) and the shared access to the stories might have influenced the mothers’ referential choice. The adults in HHRL and WHZ, however, performed the task in the absence of shared knowledge. Recall that some of our mothers were non-native speakers of Mandarin/English. It is possible that the kind of Mandarin/English they spoke differed from that of native speakers. We leave this for future research.
