# Supplementary material for: Reference production in Mandarin–English bilingual preschoolers: Linguistic, input, and cognitive factors
Source: Front Psychol. 2022 Sep 29;13:897031. doi: 10.3389/fpsyg.2022.897031 (PMC9559801; doi:10.3389/fpsyg.2022.897031)
Supplement: Supplementary file 1 [file Data_Sheet_1.PDF]

Coding Rubrics

Referential forms

Referential forms	Mandarin	English
Nominals	**+ [Num-Cl-N]**<br>e.g., *Lai le yi zhi mao*. ‘There comes a cat.’<br>*Liang zhi xiaoniao hen haipa*. ‘The two birds are very scared.’	**+ [Indef./Def./Num. determiner-NP]**<br>e.g., *There comes a cat. The birds are scared. There are two birds.*
	**+ Bare noun**<br>e.g., *You mao*. ‘There is a cat. / There are cats.’<br>*Huli hen e*. ‘The fox is very hungry.’	**+ No determiner singular N (used as a proper noun)**<br>e.g., *Cat is so naughty.*
	**+ Demonstrative NP**<br>e.g., *na zhi yang* ‘that goat’	**+ Demonstrative NP**<br>e.g., *that goat*
	**+ Kinship term**<br>e.g., *mama* ‘Mommy’	**+ Kinship term**<br>e.g., *Mommy*
	**+ Complex NP containing a possessor, a relative clause, or adjectival modification**<br>e.g., *ta (de) mama* ‘her mother’, *diaojin shuili de yang* ‘the goat who just dropped into the water’, *hei de niao* “the black bird”	**+ Complex NP containing a possessor, a relative clause, or adjectival modification**<br>e.g., *her babies, the goat who just dropped into the water, little birds*
Pronominals	**+ Personal pronoun**<br>e.g., *ta* ‘it’	**+ Personal pronoun**<br>e.g., *it*
	**+ Demonstrative**<br>e.g., *zhe(ge)* ‘this’, *na(ge)* ‘that’	**+ Demonstrative**<br>e.g., *this, that*
Null forms	**+ Null subject/object**<br>e.g., *zhuadao le*. ‘[It] got [the bird].’	**– Null subject/object**<br>e.g., *[*It*] *got* [*the bird*].
Others	N/A	**+ nonspecific lexical items**<br>e.g., *someone*

Notes: ‘+” = allowed, “–” = disallowed, “*” = ungrammatical, “[]” = lexically unrealized

Syntactic position of first mentions

**Syntactic position**	**Examples (target referential form underlined)**		
	**Mandarin**		**English**
**Preverbal**	Canonical subject	*Zai shuxia de xiaomao kanjian le shushing de xiaoniao* ‘A cat under the tree saw the birds in the tree.’	*The cat saw the birds.*
	NP in *ba*-construction	*Xiang yao ba xiaoyang chi diao*. ‘[The fox] wanted to have the little goat eaten up.’	N/A
**Postverbal**	Canonical object	*Ta zhengzai wei ta de niaobaobao*. ‘She’s feeding her baby birds.’	*The cat caught a bird.*
	SV inversion	*Shu xia ye lai le yi zhi xiaohuanggou*. ‘Under the tree there comes a little yellow dog.’	*Along came a cat.*
	NP after *you* (there be) in existential construction	*You yizhi huli lai le*. ‘There is a fox coming.’	*There is a cat looking at his baby.*
**Unanalysable**	NP-only	*Liangzhi xiaoyang*. ‘Two baby goats.’	*A cat.*
	Verbal predicate missing	N/A	*The three little goats the grass on there.*<br><br>*The small sheep in the water.*

Discourse functions

Discourse function was coded largely following Serratrice (2007), with reference to Colozzo and Whitely (2014). The unit of analysis is “clause” defined by the presence of a verbal predicate (Serratrice, 2007). The verbal predicates are mainly verbs and may include adjectives in Mandarin.

Introduction: the very first mention of the referent (including NP-only).

Reintroduction: reference involving topic shift across adjacent clauses.
To be coded as reintroduction, a referential form must meet one of the following criteria: (i) it is a subject/object argument referring to a previously identified referent which has not been mentioned in the immediately preceding clause; (ii) it is a subject argument and has been mentioned in the adjacent clause as a non-subject (e.g., an object, or an adjunct); or (iii) the reference shifts from two or more characters together to only one of these characters (and vice versa). All subsequent mentions other than reintroduction were coded as maintenance.

Sample coding of discourse functions in English and Mandarin Chinese is provided below.

Sample coding of discourse functions in English (target referential form underlined)

Unit ID	Clause	Referent	Function
1	there is <u>a goat</u> in the pond .	Baby goat1	Introduction
2	<the other> [//] <u>the goat in the pond</u> ask for the other goat for help .	Baby goat1	Reintroduction
2	<the other> [//] the goat in the pond ask for <u>the other goat</u> for help .	Mommy goat	Introduction
3	<u>the other goat</u> push him out of the [/] the pond	Mommy goat	Reintroduction
3	the other goat push <u>him</u> out of the [/] the pond	Baby goat1	Maintenance
4	when <the the goat> [//] <u>the other goat</u> was eating grass .	Baby goat2	Introduction
5	<u>the wolf</u> wanted to eat the [/] the one in the pond .	Fox	Introduction
5	the wolf wanted to eat the [/] <u>the one in the pond</u> .	Baby goat1	Reintroduction
6	so then the [/] <u>the wolf</u> come	Fox	Maintenance
7	and ϕ chase the [/] the goat .	Fox	Maintenance
7	and ϕ chase the [/] <u>the goat</u> .	Baby goat2	Reintroduction
8	and ϕ pull one of the goat's leg .	Fox	Maintenance
9	<u>he</u> almost fell	Baby goat2	Reintroduction
10	when <u>two of them</u> was drinking water .	Baby goat1 and Mommy goat	Reintroduction
11	<u>the bird on the tree</u> was so angry	Crow	Introduction
12	that he [//] <u>the bird</u> flew to the [/] the wolf	Crow	Maintenance
12	that he [//] the bird flew to the [/] <u>the wolf</u>	Fox	Reintroduction
13	and ϕ bite his [/] his tail .	Crow	Maintenance
14	then the [/] the wolf , I [/] I mean , <u>the goat</u> was so happen [//] happy .	Baby goat2	Reintroduction

Notes: Units with more than one referential form are repeated as many times as the number of referential forms they contain. ϕ = null form. Transcription follows the CHAT-format (MacWhinney, 2000): "[//]" indicates self-correction and "[/]" self-repetition.

Sample coding of discourse functions in Mandarin Chinese (target referential form underlined)

Unit ID	Clause	Referent	Function
1	有 一 天 , 一 [/] 一 只 小 [/] 小 山羊 在 [/] 在 草地 上面 吃 草 .	Baby goat2	Introduction
2	然后 呢 , 一 只 山羊 不小心 [: 一不小心] 跑 到 水 里面 .	Baby goat1	Introduction
3	然后 那个 大 一点 的 山羊 去 救 那个 小 山羊 .	Mommy goat	Introduction
3	然后 那个 大 一点 的 山羊 去 救 那个 小 山羊 .	Baby goat1	Maintenance
4	突然 , 一 个 狐狸 看到 那个 [/] 那个 小 山羊 在 吃 草 .	Fox	Introduction
4	突然 , 一 个 狐狸 看到 那个 [/] 那个 小 山羊 在 吃 草 .	Baby goat2	Reintroduction
5	它 想 要 抓住 那个 山羊 .	Fox	Maintenance
5	它 想 要 抓住 那个 山羊 .	Baby goat2	Maintenance
6	然后 [/] 然后 呢 , 那个 大 山羊 看到 它 在 吃 草 .	Mommy goat	Reintroduction
6	然后 [/] 然后 呢 , 那个 大 山羊 看到 它 在 吃 草 .	Baby goat1	Reintroduction
7	然后 呢 , 那个 小 山羊 刚刚 在 吃 草 的 时候	Baby goat2	Reintroduction
8	ɸ 看到 一 只 狐狸 要 抓 它 xxx .	Baby goat2	Maintenance
8	ɸ 看到 一 只 狐狸 要 抓 它 xxx .	Fox	Reintroduction
8	ɸ 看到 一 只 狐狸 要 抓 它 xxx .	Baby goat2	Maintenance
9	它 就 要 快 跑 走 .	Baby goat2	Maintenance
10	但是 呢 , 那个 狐狸 已经 抓住 它 的 脚 了 .	Fox	Reintroduction
11	然后 一 只 鸟 看见 .	Crow	Introduction
12	它 想 要 救 那个 羊 .	Crow	Maintenance
12	它 想 要 救 那个 羊 .	Baby goat2	Reintroduction
13	然后 <那 一> [//] 那个 鸟 快快 把 [/] 把 狐狸 的 [/] 的 尾巴 给 咬 住 .	Crow	Maintenance
14	然后 那个 狐狸 它 觉得 很 痛 .	Fox	Reintroduction
14	然后 那个 狐狸 它 觉得 很 痛 .	Fox	Maintenance
15	然后 <它 就> [/] 它 就 把 [/] 把 手 松 掉 .	Fox	Maintenance
16	然后 那个 [?] 山羊 看到 它 .	--	--
16	然后 那个 [?] 山羊 看到 它 .	Fox	Maintenance

Sample coding of discourse functions in Mandarin Chinese (*continued*)

Unit ID	Clause	Referent	Function
17	它 说 +"/.	Baby goat2	Maintenance
17	+" 嗯 ?	--	--
18	然后 呢 , 它 [/] 它 们 就 被 救 了 .	Baby goat1&2 and Mommy goat	Reintroduction
19	然后 那个 大 [/] 大 一点 的 山羊 xxx 它 们 .	Mommy goat	Reintroduction
19	然后 那个 大 [/] 大 一点 的 山羊 xxx 它 们 .	Baby goat1&2	Reintroduction
20	然后 <那 只 鸟> [///] 狐狸 跑 走 的 时候 ,	Fox	Reintroduction
21	那 只 鸟 还 在 追 那个 狐狸 .	Crow	Reintroduction
21	那 只 鸟 还 在 追 那个 狐狸 .	Fox	Maintenance

Notes: Units with more than one referential form are repeated as many times as the number of referential forms they contain. ϕ = null form. Transcription follows the CHAT-format (MacWhinney, 2000): “[//]” indicates self-correction, “[/]” self-repetition, “xxx” unintelligible words, “[?]” unclear, “+"/” start of direct speech from next line, “+"” direct speech, and “[///]” false start.
